# Is pregnancy status being assessed within women's secure services?

**DOI:** 10.1192/bjo.2021.902

**Published:** 2021-06-18

**Authors:** Jeremy Rampling, Shay-Anne Pantall, Hannah Woodman

**Affiliations:** 1 Birmingham and Solihull Mental Health NHS Foundation Trust; 2 University of Birmingham, Medical School

## Abstract

**Aims:**

To establish rates of pregnancy testing on admission of women within a blended secure service.

**Background:**

Women with psychiatric illness are known to be at increased risk of pregnancy, often due to engagement in risky sexual behaviours such as having a higher numbers of sexual partners and engaging in sexual activity whilst under the influence of drugs or alcohol. Awareness of pregnancy at the point of admission to psychiatric hospital would inform ongoing care plans to manage the pregnancy in the safest, least restrictive environment and inform future prescribing decisions, to minimise the risk of teratogenicity associated with some psychotropic medications. Ardenleigh in Birmingham is a blended female secure unit. No pregnancy screening guidelines for this population currently exist. This audit sought to establish current rates of pregnancy testing at the point of admission with a view to developing future guidelines.

**Method:**

A retrospective case note audit of electronic records of all patients admitted to Ardenleigh blended women's service as of 1st September 2019 (n = 26). The expected standard for pregnancy testing within one month of admission was set as 100%.

**Result:**

Key results include:

- The majority of patients (67%) were aged under 35 years (range 20–56). The most common ethnicities were Caucasian (42%) and African-Caribbean (38%). Almost half (46%) had a primary diagnosis of paranoid schizophrenia.
- Two women were known to be pregnant at the point of admission. Only 54% of women with an unknown pregnancy status were screened for pregnancy within one month of admission.
- Rates of screening were particularly poor in women aged under 25 years (43%) and between 36 and 45 (0%).
- Women not screened for pregnancy were typically admitted from other hospital settings, including AWA services (27%) or other medium secure units (55%). 2 women admitted from prison were not tested (29%)
- Of those tested, the majority were checked using urine hCG (92%).

None of the women tested were found to be pregnant.

**Conclusion:**

Overall pregnancy testing on admission to the unit was poor, with only 54% of service users screened. Less than 100% compliance could result in serious consequences for both the woman and unborn baby if a pregnancy is not discovered. Updating the admission checklist for Ardenleigh to include pregnancy testing may prove beneficial. It is recommended that a re-audit is completed 6 months following checklist introduction.

